# Human milk-derived versus bovine milk-derived fortifier use in very low birth weight infants: growth and vitamin D status

**DOI:** 10.3389/fped.2024.1354683

**Published:** 2024-02-19

**Authors:** Emmanuelle Lavassani, Kate A. Tauber, Jennifer B. Cerone, Jennifer Ludke, Upender K. Munshi

**Affiliations:** ^1^ Department of Pediatrics, Duke University School of Medicine, Durham, NC, United States; ^2^ Department of Pediatrics, Albany Medical Center, Albany, NY, United States

**Keywords:** human milk-derived fortifier, bovine milk-derived fortifier, preterm infant nutrition, breast milk fortification, 25OH vitamin D

## Abstract

**Background:**

Human milk-derived fortifier (HMDF) coupled with human milk feeding in extremely premature infants reduces the adverse outcome risks of early exposure to bovine milk ingredients but may not provide enough nutrients for adequate catch-up growth compared with bovine milk-derived fortifier (BMDF).

**Objective:**

This study aims to compare HMDF and BMDF effects on growth parameters and serum 25-hydroxy vitamin D (25OHD) levels in preterm very low birth weight (VLBW) infants during the first 8 weeks of life.

**Methods:**

We present a retrospective chart review of inpatient VLBW infants with birth weight <1,500 g and gestational age <32 completed weeks who received either their mother’s milk or donor breast human milk fortified with HMDF or BMDF for the first 8 weeks. Weight, head circumference, length gain, and 25OHD level were calculated at 4 and 8 weeks of age.

**Results:**

A total of 139 VLBW infants (91 HMDF + 48 BMDF) received fortified human milk without any supplemental premature formula from birth to 4 weeks of age, of whom 44 (37 HMDF + 7 BMDF) continued until 8 weeks of age. There was no statistically significant difference in the growth parameters between the two groups at 4 and 8 weeks of age. Serum 25OHD level in the HMDF group was significantly higher compared with that in the BMDF group at 4 weeks of age despite receiving lower vitamin D supplementation.

**Conclusion:**

Similar gain in growth parameters in HMDF and BMDF groups at 4 and 8 weeks of age was observed, suggesting that HMDF provides adequate nutrients for growth in VLBW infants. A higher 25OHD level in HMDF may suggest better absorption.

## Introduction

Premature infants with <32 weeks gestational age and/or <1,500 g birth weight are deprived of a robust protein and mineral accretion that is achieved in the third trimester. Unfortified breast milk is not able to meet their nutritional demands after birth due to a lack of fetal accretion and rapid catch-up growth. Bovine milk-derived fortifier (BMDF) has been the standard of care for breast milk fortification for very low birth weight (VLBW) infants (<1,500 g) born prematurely until the introduction of human milk-derived fortifier (HMDF) (1–3). HMDF provides an opportunity for an exclusive human milk diet and has been reported to reduce the parenteral nutrition period, hospitalization duration, and complications associated with early exposure to bovine milk ingredients in VLBW infants, particularly necrotizing enterocolitis (NEC) (4, 5). Despite the decrease in the risk for adverse events associated with HMDF, concerns have been raised about whether HMDF provides sufficient nutrients to support the catch-up growth of VLBW infants (6). Nutrient deficiency in preterm infants fed breast milk alone can compromise growth, limit bone health, and increase susceptibility to osteopenia of prematurity. Vitamin D enhances gastrointestinal calcium absorption and is a key contributor to overall bone health (7). We reported that 80% of VLBW infants had deficient or insufficient serum 25-hydroxy vitamin D (25OHD) serum levels at 4 weeks of age before oral supplementation was started (8). The American Academy of Pediatrics (AAP) recommends all VLBW infants receive a daily intake of 200–400 international units (IU) of vitamin D until weight >1,500 g, at which time they should receive 400 IU (7). Now more institutions have switched to HMDF with vitamin D supplementation, but there are only a few studies that have compared the two types of human milk fortifiers in early life. This study was undertaken to fill the knowledge gap in the current literature comparing the effects of HMDF and BMDF on growth and vitamin D levels in premature VLBW infants in the first 2 months of life.

## Methods

We present an IRB-approved retrospective chart review of infants, with a birth weight of <1,500 g and/or gestational age of <32 completed weeks, who were admitted to a level 4 neonatal intensive care unit (NICU) within 24 h of birth.

### Study population

This study enrolled preterm infants with <32 weeks gestational age and/or birth weight of <1,500 g who were admitted to level 4 NICU from 2015 to 2020 and received human milk (mother's or donor) fortified with BMDF or HMDF.

### Inclusion criteria

We included all infants with <32 weeks gestation and/or birth weight of <1,500 g who initially received parenteral nutrition and human (mother's or donor) milk that was fortified with BMDF or HMDF and subsequently switched to receiving all enteral feeds of human milk (mother's or donor) fortified with BMDF or HMDF for the first 8 weeks of life.

### Exclusion criteria

All infants with <32 weeks gestational age and/or birth weight <1,500 g who received any preterm formula with or without partial human milk were excluded. Other exclusion criteria included infants who were discharged, were transferred out of the NICU prior to 8 weeks of age, died within 1 month of age, had NEC or gastrointestinal surgery, failed to achieve full enteral feeds, did not receive breast milk fortification, had lower serum 25OHD levels, had congenital anomalies of the gastrointestinal system, and had major chromosomal abnormalities. VLBW infants who were considered small for gestational age (SGA) based on their gestation and birth weight (<10th percentile) and large for gestational age (LGA) (>90% percentile) were also excluded from analysis due to their differences in metabolic demands from infants who were considered appropriate for gestation age (AGA) (9).

### Feeding fortification

Infants receiving human milk (mother’s or donor) feed fortified with BMDF or HMDF were followed up until 8 weeks of age using the same type of fortifier and were stratified into two groups, namely, the BMDF and HMDF groups, based on the type of fortifier received. BMDF was the primary breast milk fortifier used in our NICU for VLBW infants until the end of 2018, at which time HMDF was introduced as the preferred fortifier from 2019 onward. The feeding protocols in the NICU remained unchange between 2015 and 2020, except for the substitution of BMDF by HMDF as the preferred fortification. The macro- and micronutrient composition of human milk after HMDF and BMDF fortification had minor important differences that were calculated by our clinical nutritionist from the information provided by the manufacturer in a table form as per 100 ml of reconstituted feed (see Supplementary Table S1). All infants continued to receive only one kind of fortification until they were >34 weeks and/or >1,500 g, at which time HMDF-fortified infants were transitioned to BMDF-fortified milk and or preterm formula when human milk was low in quantity or not available. This policy of transitioning HMDF infants beyond 34 weeks postmenstrual age to BMDF of human milk or preterm formula was done because of the higher cost and lower availability of HMDF and lower risk of feeding intolerance and NEC in more mature infants. All VLBW infants, after receiving enteral feeds at 120 ml/kg/day, were given supplemental oral vitamin D_3_ (400 IU/ml cholecalciferol, which is equal to 10 ng/ml) as per AAP recommendations (10).

### Laboratory tests

Serum calcium, phosphorus, alkaline phosphatase, and 25OH vitamin D grouped as nutritional labs were done routinely ∼4 and 8 weeks of age.

Serum 25OHD assay was performed at 4 and 8 weeks of age using DiaSorin Liaison Immunoassay (DiaSorrin Inc., Stillwater, MN, USA). For operational definitions for this study, serum 25OHD levels were considered deficient at <20 ng/ml, insufficient at 20–29 ng/ml, normal at 30–60 ng/ml, high at 61–100 ng/ml, and very high at >100 ng/ml (8, 10). Elevated alkaline phosphatase was defined as >600 IU, hypercalcemia as serum calcium >11 mg/dl, hyperphosphatemia as serum phosphate >8 mg/dl, and hypophosphatemia as serum phosphate >4 mg/dl. Vitamin D supplementation was calculated for each infant as the sum of the vitamin D drops supplemented from the milk fortifier.

### Data collection

Data collected included growth metrics [weight, length, and head circumference (HC)], demographic information, fortification type, breast milk type (MBM vs. DBM), days achieved and gestational age when enteral intake reached 120 ml/kg/day (with discontinuation of intravenous nutrition), calculated calorie and protein intake, vitamin D supplementation, and serum 25OHD, calcium, phosphorus, and alkaline phosphatase levels at 4 and 8 weeks of age. Enteral feeds were advanced by 20 ml/kg/day–160 ml/kg/day, which was full enteral feeds per our feeding protocol. Laboratory data were collected as close as possible to the 4 and 8 weeks of age, with a maximum range of 7 days from the target date. Growth metrics were collected within 1 week of the dates of 4 and 8 weeks of age unless the patient was discharged or transferred out, before 4 weeks meeting the exclusion criteria.

The type of human milk fortification and caloric, protein, and vitamin D intake were calculated from feeding orders at 4 and 8 weeks of age.

### Statistical analysis

Statistical analysis was conducted utilizing Microsoft Excel and GraphPad InStat Prism 9.0. All data sets were assessed for distribution to determine analysis via parametric or non-parametric methods using the Shapiro–Wilk normality test. In GraphPad InStat Prism, two-tailed unpaired *t*-tests were used for parametric numerical datasets, Mann–Whitney *U* tests were used for non-parametric numerical datasets, and Fisher's exact tests were used to analyze categorical variables. Significance was set at *P *< 0.05.

## Results

**Figure 1:**
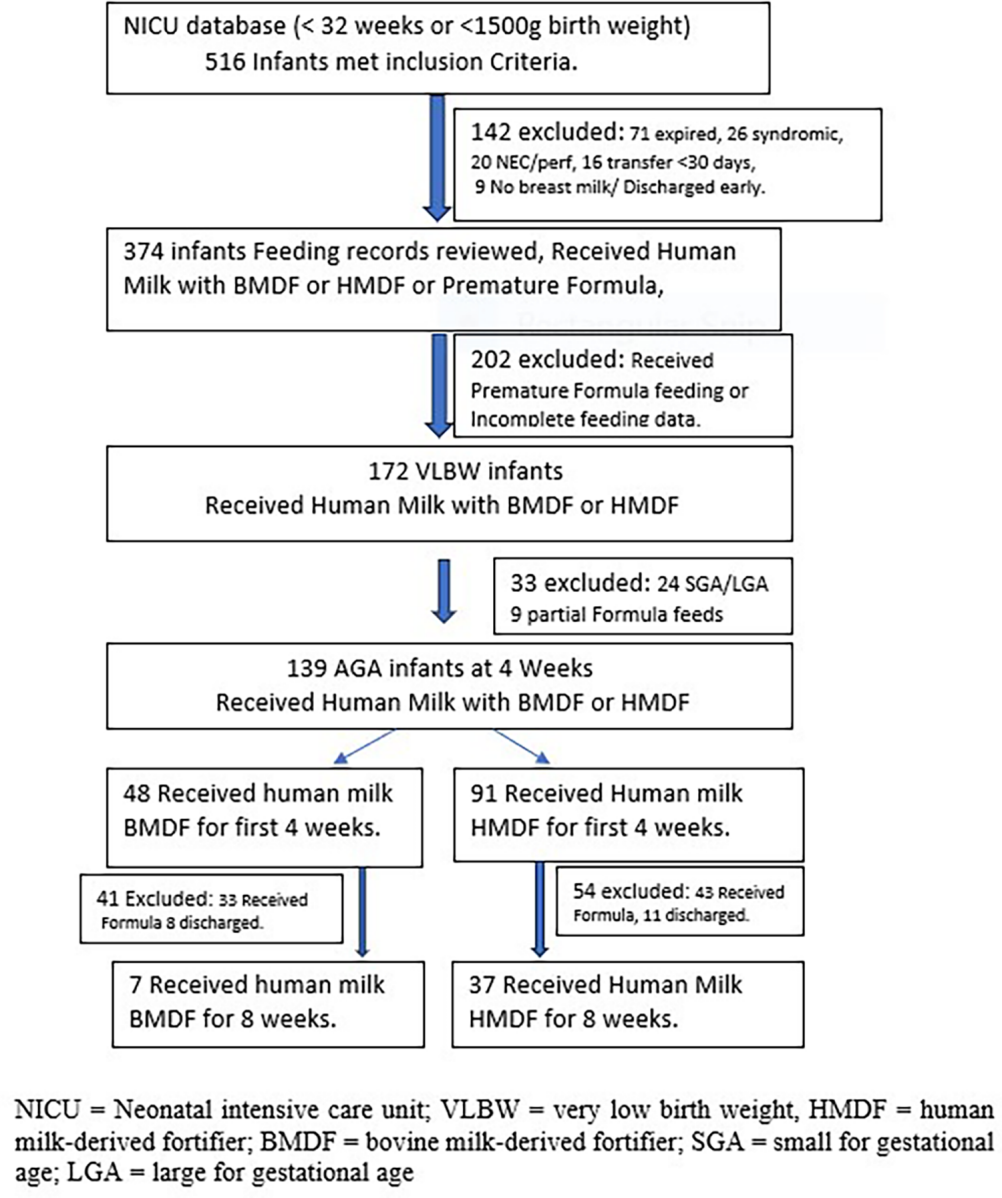
Infant flowchart diagram showing screening and exclusion of infants to final enrollment of 139 infants at 4 weeks and 44 infants at 8 weeks of age.

A total of 516 infants were screened for eligibility, of whom 139 infants (91 HMDF + 48 BMDF) at 4 weeks of age and 44 infants (37 HMDF + 7 BMDF) at 8 weeks of age met the inclusion criteria for analysis (Figure 1).

### Demographic characteristics

The HMDF and BMDF groups had similar demographic characteristics with no statistical difference in gestational age, birth weight, birth length, and birth HC (Table 1). There was no statistically significant difference in gestational age and days to reach enteral feeds of 120 ml/kg/day or percentage of infants receiving >50% donor human milk between the two groups. The infants receiving HMDF reached all enteral feeds in 10.5 ± 6.1 days, whereas the infants receiving BMDF reached all enteral feeds in 11.5 ± 6.9 days, *P *= 0.46. The average gestational age (±SD) at which all enteral feeds were achieved was also similar (HMDF group, 29.6 ± 2.8 weeks; BMDF group, 29.0 ± 1.6 weeks; *P *= 0.15).

**Table 1:** Demographics of the HMDF and BMDF groups receiving human milk and fortified until 4 weeks of age.

Total *N* = 139	HMDF (*n* = 91)	BMDF (*n* = 48)	*P*
Gestational age, week, (mean ± SD)	27.7 ± 1.9	27.4 ± 2.0	0.41
Birth weight, g, (mean ± SD)	1,044 ± 254	993 ± 213	0.22
Birth length, cm, (mean ± SD)	36.0 ± 3.2	35.7 ± 3.2	0.55
Birth HC, cm, (mean ± SD)	24.4 ± 1.9	24.7 ± 1.9	0.32
Female sex, *n*, (%)	49 (54%)	25 (52%)	0.86
Receiving >50% donor milk	32 (35%)	10 (26%)	0.09
Days to all enteral feeds, days, (mean ± SD)	10.5 ± 6.1	11.5 ± 6.9	0.46
Gestational age at all enteral feeds, week, (mean ± SD)	29.6 ± 2.8	29.0 ± 1.6	0.15

HMDF, human milk-derived fortifier; BMDF, bovine milk-derived fortifier; SD, standard deviation; HC, head circumference. Full enteral feeds were achieved at 120 ml/kg/day.

### Growth parameters

Weight, HC, and length gain from birth were compared at 4 and 8 weeks of age, and no statistically significant difference was found between the BMDF and HMDF groups (Table 2). When comparing protein intake between the groups, we found that both received similar protein intake at 4 weeks of age, with an average protein intake of 4.1 ± 0.5 g/kg in the HMDF group and 4.0 ± 0.3 in the BMDF group (*P* = 0.22). Similarly, at 8 weeks of age, the HMDF group received 4.2 ± 0.7 g/kg protein, whereas the BMDF group received 4.3 ± 0.2 g/kg (*P* = 0.75). The caloric intake in the HMDF group at 4 weeks of age was higher than that of the BMDF group with average values of 151 ± 15 kcal/kg and 125 ± 9 kcal/kg, respectively (*P* < 0.0001). No significant difference was found in caloric intake between the two groups at 8 weeks of age, with HMDF receiving 144 ± 18 kcal/kg and BMDF receiving 131 ± 6 kcal/kg (*P* = 0.75).

**Table 2:** Growth parameters, including weight, length, and head circumference gain, of the HMDF and BMDF groups at 4 and 8 weeks of age.

	Four weeks			Eight weeks		
	HMDF (*n* = 91)	BMDF (*n* = 48)	*P*	HMDF (*n* = 37)	BMDF (*n* = 7)	*P*
Weight gain, g, (mean ± SD)	377 ± 152	342 ± 161	0.21	1,027 ± 284	1,056 ± 236	0.87
Length gain, cm, (mean ± SD)	2.8 ± 1.6	3.1 ± 1.8	0.33	7.0 ± 1.7	8.6 ± 3.8	0.07
HC gain, cm, (mean ± SD)	2.3 ± 1.0	2.3 ± 1.0	0.76	5.9 ± 1.5	5.8 ± 1.5	0.92

HMDF, human milk-derived fortifier; BMDF, bovine milk-derived fortifier; SD, standard deviation; HC, head circumference, 250HD, 25-hydroxy vitamin D. Laboratory values for some of the subjects were not available for analysis.

### Laboratory results

**Figure 2:**
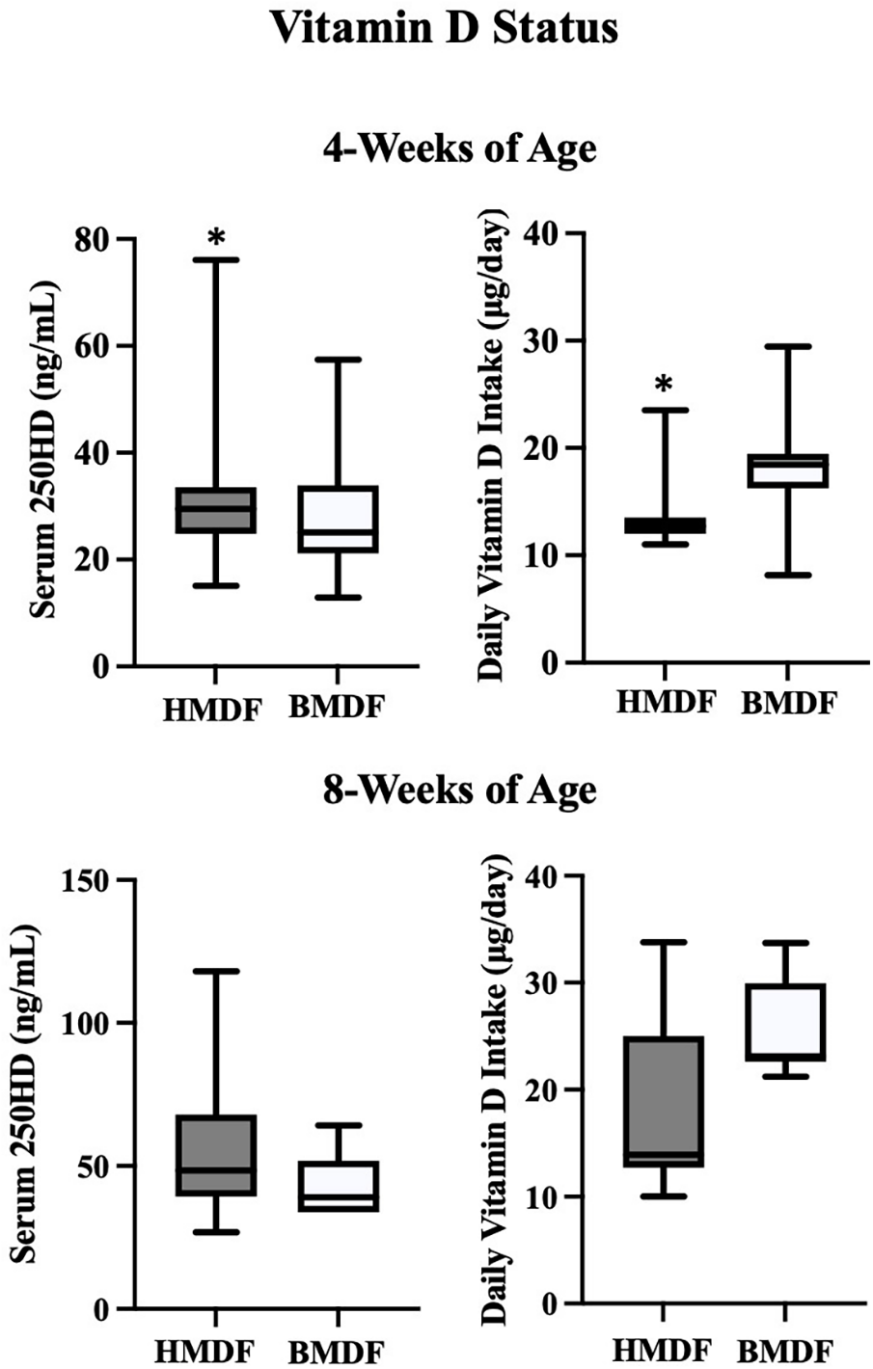
Serum 25 OHD levels and vitamin D intake (diet + supplement) in HMDF and BMDF infants at 4 weeks and 8 weeks of age.

The average serum 25OHD level at 4 weeks of age was 30.6 ± 9.0 ng/ml in the HMDF group and 28.2 ± 10.9 ng/ml in the BMDF group (*P* = 0.02). At 4 weeks of age, the HMDF group received 13.7 ± 3.3 μg (532 ± 132 IU) of vitamin D per day, whereas that for the BMDF group was 18.3 ± 3.7 μg (732 ± 148 IU) per day (<0.0001) (Figure 2). The average serum 25OHD level in the HMDF group at 8 weeks of age was 53.9 ± 20.2 ng/ml, whereas that for the BMDF group was 42.9 ± 11.7 ng/ml. At 8 weeks of age, the HMDF group received 18.5 ± 7.0 μg (740 ± 280 IU) of vitamin D per day, whereas the BMDF group received 25.5 ± 4.8 μg (1,020 ± 192 IU) of vitamin D per day. There was an increase in the average serum 25OHD levels within each group from 4 to 8 weeks of age, with 76.1% for the HMDF group, receiving a lower vitamin D intake, and 52.1% for the BMDF group.

During the study period, out of the 139 infants included who received fortified human milk, 7 (5%) infants were diagnosed with NEC after the first 4 weeks; the HMDF group had 4 infants (2 had surgical intervention and both survived), and the BMDF group had 3 infants (2 had surgical intervention, and both died).

There was no statistically significant difference in serum calcium, phosphate, or alkaline phosphatase levels in either group at 4 and 8 weeks of age. In the HMDF group at 4 weeks of age, the mean serum calcium was 9.9 ± 0.4 mg/dl, whereas that for the BMDF group was 9.8 ± 0.5 mg/dl (*P* = 0.94). The HMDF group at 4 weeks of age had an average serum phosphorus level of 6.4 ± 0.8 mg/dl, whereas the BMDF group had 6.6 ± 1.1 mg/dl (*P* = 0.26). The HMDF group at 4 weeks of age had a serum alkaline phosphatase level of 360 ± 125 IU, whereas the BMDF group had 359 ± 137 IU (*P* = 0.54). At 8 weeks of age, the HMDF group had a mean serum calcium level of 9.9 ± 0.5, whereas the BMDF group had 10.1 ± 0.4 (*P* = 0.55). The average serum phosphate level in the HMDF group at 8 weeks of age was 6.1 ± 0.8 mg/dl, whereas the BMDF group had 6.5 ± 0.6 mg/dl (*P* = 0.16). The HMDF group at 8 weeks of age had a mean serum alkaline phosphatase level of 323 ± 89 mg/dl, whereas the BMDF group had 315 ± 70 mg/dl (*P* = 0.82). In the HMDF group, six VLBW infants at 4 weeks of age had elevated alkaline phosphatase levels (>600 IU). Following up at 8 weeks of age, no patients in the HDMF group had elevated levels. Similarly, one VLBW infant in the BMDF group had elevated alkaline phosphatase level at 4 weeks of age, and none had elevated levels at 8 weeks of age.

## Discussion

We found no statistical differences in weight, length, and HC growth between the HMDF and BMDF groups at 4 and 8 weeks of age. The lack of difference in growth parameters supports a conclusion of non-inferiority between the impact of the two fortifiers on VLBW growth in AGA infants. O'Connor et al.’s (5) randomized controlled trial comparing growth outcomes in HMDF vs. BMDF in preterm infants similarly found no differences in weight, length, or HC growth between the groups. A study by Fleig et al. (11) reported no change in the growth parameters in SGA VLBW infants who received HMDF vs. BMDF except for the significantly increased length gain since birth in the HMDF group. Additionally, Fleig et al. found significantly decreased rates of NEC, surgical NEC, and late-onset sepsis in SGA VLBW infants who received HMDF in comparison with those of infants who received milk fortified with BMDF (11). Our findings, along with those from these studies, confirm that HMDF provides sufficient protein and nutrients to support VLBW infant growth.

Our study shows that VLBW infants receiving HMDF at 4 weeks of age had significantly higher serum 25OHD levels than those in the BMDF group, despite receiving less vitamin D supplementation than the BMDF infants who had higher vitamin D intake from their fortified feeding. At 4 weeks of age, the BMDF group had overall vitamin D insufficiency, whereas the HMDF group had just achieved levels in the normal range. At 8 weeks of age, both groups met sufficient vitamin D status with trending higher levels of 25OHD, despite the lower intake of vitamin D in the HMDF group, although this did not reach statistical significance, probably due to the low number of VLBW infants who remained only on human milk without any supplementation of premature formula. The greater increase in serum 25OHD levels within the HMDF group while receiving a lower amount of supplemental Vitamin D from 4 to 8 weeks of age compared with those in the BMDF group similarly suggests that this could be attributed to improved enteral absorption of vitamin D.

There is insufficient data in the current literature comparing the effects of HMDF and BMDF in terms of serum vitamin D levels, but it has been widely reported that VLBW infants are at an increased risk of vitamin D deficiency (8, 12). Adnan et al. (12) reported vitamin D deficiency or insufficiency in over 50% of the VLBW infants in their study and found that fortification with BMDF and supplementation with 200–400 IU of vitamin D was sufficient to correct for these conditions by 2–3 weeks of age and to maintain sufficient levels thereafter. Munshi et al. similarly reported correction of VLBW infant vitamin D deficiency or insufficiency following oral vitamin D supplementation but recommended the use of serum 25OHD levels to monitor response and safety as the increase in vitamin D levels was not consistent with dosing (8).

Elevated alkaline phosphatase is a biomarker indicative of metabolic bone disease (13). When reviewing markers for bone density, such as alkaline phosphatase levels, and looking for clinical or radiographic evidence of rickets, Premkumar et al. in their systematic review found no difference in outcomes for preterm infants receiving milk fortified with HMDF vs. BMDF (3). We also did not find any significant difference in the number of infants with elevated alkaline phosphatase in either group at 4 or 8 weeks of age, suggesting that dietary mineral supplements of calcium and phosphate were adequate in both groups. Additionally, the decreased mortality rate of NEC in the HMDF group compared with that of the BMDF group supports the findings from previous studies, which reported that an exclusive human diet with HMDF decreases the risk of adverse NEC outcomes compared with BMDF (4, 11, 14).

Our study has a few limitations: it is a retrospective chart review study, and the number of subjects who fit the inclusion criteria for analysis is low, particularly in the BMDF cohort. Many patients were discharged between the 4 and 8 week timepoints or transitioned to preterm formula, both of which contributed to a decreased number of subjects for data analysis. Additionally, data on maternal ethnic race and serum 25OHD levels were not available for the assessment of maternal vitamin D status at the time of delivery. Maternal race and intake of vitamins during pregnancy are known to contribute to infant vitamin D status at birth (15).

## Conclusions

Growth parameters were similar between the groups at both 4 and 8 weeks of age, suggesting that HMDF provides adequate nutrients for growth in AGA VLBW infants. The VLBW infants who received exclusively HMDF had higher serum 25OHD levels at 4 weeks of age than those in VLBW infants who received BMDF, despite the HMDF group receiving a lower daily intake of vitamin D. The mechanism of a higher 25OHD level in the HMDF group is not clear at this point and needs to be further studied. We suggest that the HMDF group may have higher bioavailability or better absorption of vitamin D.

## Data Availability

The original contributions presented in the study are included in the article/**Supplementary Material**; further inquiries can be directed to the corresponding author.

## Supplementary material

Supplementary Table S1 Macro- and micro-nutrient comparison of 100 ml reconstituted feed by HMDF and BMDF fortification, compared with unfortified Preterm Milk as reference.

## References

[1] Hopperton KE O'Connor DL Bando N Conway AM Ng DV Kiss A Nutrient enrichment of human milk with human and bovine milk-based fortifiers for infants born <1250g: 18-month neurodevelopment follow-up of a randomized clinical trial. Curr Dev Nutr. (2019) 3:nzz129. 10.1093/cdn/nzz129 32154499 PMC7053578

[2] Vogiatzi MG Jacobson-Dickman E Deboer MD. Vitamin D supplementation and risk of toxicity in pediatrics: a review of current literature. J Clin Endocrinol Metab. (2014) 99:1132–41. 10.1210/jc.2013-3655 24456284

[3] Premkumar MH Pammi M Suresh G. Human milk-derived fortifier versus bovine milk-derived fortifier for prevention of mortality and morbidity in preterm neonates. Cochrane Database Syst Rev. (2019) 2019:1–31. 10.1002/14651858.CD013145.pub2 PMC6837687 31697857

[4] Mizuno K Shimizu T Ida S Ito S Inokuchi M Ohura T Policy statement of enteral nutrition for preterm and very low birthweight infants. Pediatr Int. (2020) 62:124–7. 10.1111/ped.14067 32026585 PMC7065204

[5] O'Connor DL Kiss A Tomlinson C Bando N Kayliss A Campbell DM Nutrient enrichment of human milk with human and bovine milk-based fortifiers for infants born weighing <1250g: a randomized clinical trial. Am J Clin Nutr. (2018) 108:108–16. 10.1093/ajcn/nqy067 29878061

[6] Arslanoglu S Boquien CY King C Lamireau D Tonetto P Barnett D. Fortification of human milk for preterm infants: update and recommendations of the European Milk Bank Association (EMBA) Working Group on Human Milk Fortification. Front Pediatr. (2019) 7:76. 10.3389/fped.2019.00076 30968003 PMC6439523

[7] Abrams SA, Nutrition. Co. Calcium and vitamin D requirements of enterally fed preterm infants. Pediatrics. (2013) 131:e1676–83. 10.1542/peds.2013-0420 23629620

[8] Munshi UK Graziano PD Meunier K Ludke J Rios A. Serum 25 hydroxy vitamin D levels in very low birth weight infants receiving oral vitamin D supplementation. J Pediatr Gastroenterol Nutr. (2018) 66:676–9. 10.1097/MPG.0000000000001831 29112088

[9] Larsson A Ottosson P Törnqvist C Olhager E. Body composition and growth in full-term small for gestational age and large for gestational age Swedish infants assessed with air displacement plethysmography at birth and at 3–4 months of age. PLoS One. (2019) 14:e0207978. 10.1371/journal.ponc.0207978 31091240 PMC6519902

[10] Munshi UK Graziano PD Meunier K Ludke J Rios A. Vitamin D intake in very low birth weight infants in neonatal intensive care unit. J Pediatr Gastroenterol Nutr. (2016) 63:277–9. 10.1097/MPG.0000000000001127 26825769

[11] Feig I Hagan J Lee ML Abrams SA Hawthrone KM Hair AM. Growth outcomes of small for gestational age preterm infants before and after implementation of an exclusive human milk-based diet. J Perinatol. (2021) 41:1859–64. 10.1038/s41372-021-01082-x 34012050 PMC8342303

[12] Adan M Wu SY Khilfeh M Davis V. Vitamin D status in very low birth weight infants and response to vitamin D intake during their NICU stays: a prospective cohort study. J Perinatol. (2022) 42:209–16. 10.1038/s41372-021-01238-9 34675370 PMC8528940

[13] Tinnion RJ Embleton ND. How to use alkaline phosphatase in neonatology. Arch Dis Child Educ Pract Ed. (2012) 97:157–63. 10.1136/archdischild-2012-301633 22761487

[14] Parker MG Stellwagen LM Noble L Kim JH Poindexter BB Puopolo KM. Promoting human milk and breastfeeding for the very low birth weight infant. Pediatrics. (2021) 148:1–15. 10.1542/peds.2021-054272 34635582

[15] Agarwal S Kovilam O Agrawal DK. Vitamin D and its impact on maternal-fetal outcomes in pregnancy: a critical review. Crit Rev Food Sci Nutr. (2018) 58:755–69. 10.1080/10408398.2016.1220915 27558700 PMC6056893

