# Supplementary material for: Human milk-derived versus bovine milk-derived fortifier use in very low birth weight infants: growth and vitamin D status
Source: Front Pediatr. 2024 Feb 19;12:1354683. doi: 10.3389/fped.2024.1354683 (PMC10913192; doi:10.3389/fped.2024.1354683)
Supplement: Supplementary Table S1 — Macro- and micro-nutrient comparison of 100 ml reconstituted feed by HMDF and BMDF fortification, compared with unfortified Preterm Milk as reference. [file Table1.pdf]

Supplement 1

Human Milk Fortification by Human Milk Derived fortifier (HMDF) and Bovine Milk Derived fortifier (BMDF) in Neonatal Intensive Care Unit

Nutrient content per 100 ml of fortified Feed, (Feeding ordered as ---ml/kg/Day)

**Nutrients**	**Units**	**Unfortified Preterm Human Milk**	**Fortified with Liquid HMDF***	**Fortified with Liquid BMDF****
**Calories**	Kcal	67	90.6	81
**Protein**	g	1.6	2.9	3.3
**Fats**	g	3.5	5.4	4.8
**Carbohydrates**	g	7.3	7.8	6.4
**Calcium**	mg	25	125	118
**Phosphorus**	mg	14.5	67.8	65
**Iron**	mg	0.1	0.1	1.54
**Zinc**	mg	0.4	1.5	1.11
**Vitamin D**	IU	8	8	167

Mixing Instructions per Pediatric Nutritionist in NICU

*HMDF 30ml + 70 ml Unfortified human milk = 100ml feed (26 Cal/oz)

**BMDF 5ml + 25 ml unfortified human milk = 30ml feed (24 Cal/oz)
